# Surgery on giant meningiomas in very old patients entails frequent postoperative intracranial hemorrhages and atypical histopathology

**DOI:** 10.1007/s11060-020-03693-4

**Published:** 2021-01-21

**Authors:** Roel H.L. Haeren, Ilari Rautalin, Christoph Schwartz, Miikka Korja, Mika Niemelä

**Affiliations:** 1 Department of Neurosurgery, University of Helsinki and Helsinki University Hospital, P.O. Box 266, Fin-00029-HUS, Helsinki, Finland; 2 Department of Neurosurgery, Maastricht University Medical Center, Maastricht, The Netherlands; 3 Department of Neurosurgery, University Hospital Salzburg, Paracelsus Medical University, Salzburg, Austria

**Keywords:** Surgery, Giant, Meningioma, Elderly, Atypical, Complications, Outcome

## Abstract

**Purpose:**

Surgical resection of intracranial meningiomas in patients that are 80 years old and older, i.e. very old patients, is increasingly considered. Meningiomas with a largest diameter of at least 5 cm—‘giant meningiomas’—form a distinct entity, and their surgical resection is considered more difficult and prone to complications. Here, we evaluated functional outcome, morbidity and mortality, and the prognostic value of tumor size in very old patients who underwent resection of giant supratentorial meningiomas.

**Methods:**

We retrospectively reviewed clinical and radiological data, functional performance (Karnofsky Performance Score), histopathological diagnosis and complications of very old patients who underwent surgery of a supratentorial meningioma at the Helsinki University Hospital between 2010 and 2018.

**Results:**

We identified 76 very old patients, including 28 with a giant meningioma. Patients with a giant meningioma suffered from major complications more commonly than those with a non-giant meningioma (36% vs. 17%, p = 0.06), particularly from postoperative intracranial hemorrhages (ICH). At the 1-year follow-up, functional performance and mortality rate were comparable between patients with giant meningiomas and those with non-giant meningiomas. An exceptionally high rate of giant meningiomas were diagnosed as atypical meningiomas (WHO II) at an (11 out of 28 cases).

**Conclusions:**

Giant meningioma surgery entails a high complication rate in frail, very old patients. The prevention of postoperative ICH in this specific patient group is of utmost importance. An atypical histopathology was notably frequent among very old patients with a giant meningioma, which should be taken into account when planning the surgical strategy.

**Supplementary Information:**

The online version contains supplementary material available at 10.1007/s11060-020-03693-4.

## Introduction

Meningiomas are the most common type of intracranial tumors, and the incidence rate of meningiomas increases strongly with age [1]. Up to 80–90% of intracranial meningiomas are classified as World Health Organization (WHO) type I, representing a benign tumor entity with a favorable prognosis [1]. Due to the population’s increasing life expectancy, the finding of intracranial meningiomas in very old patients, i.e. patients that are 80 years old and older, is becoming more frequent [2]. Nowadays, these very old patients with meningiomas often have a good functional status and still live independently at home, despite a known increased frailty [2–5]. Consequently, neurosurgeons are increasingly confronted with the question of whether a major surgery (i.e. intracranial tumor surgery) is justified and beneficial for these frail and very old patients [2].

Giant meningiomas, i.e. meningiomas with a largest diameter of at least 5 cm [6], form a distinct entity. Related to their size, giant meningiomas are located more frequently in eloquent areas, and have a more intricate and complex relation with neurovascular structures [6, 7]. Moreover, surgery of giant meningiomas is additionally challenging due to limited intraoperative visualization, edema, prominent vascularization and larger-sized craniotomies [6].

Since giant meningiomas are reported to occur more frequently with ageing [1, 8], and since the number of very old patients is increasing [1, 2], we studied the surgical outcome of very old patients with giant meningiomas. We hypothesized that surgery of giant meningiomas increases the risk of postoperative morbidity and mortality in very old patients. Therefore, we aimed to evaluate the (1) surgical outcome, (2) surgical morbidity and mortality, and (3) association with tumor size following the resection of giant supratentorial meningiomas in patients aged 80 years old and older.

## Methods

### Patients

This retrospective study was conducted following approval of the institutional review board of the Helsinki University Hospital.

All very old patients, i.e. aged 80 years old and older, who underwent elective surgical resection of a supratentorial meningioma at the Helsinki University Hospital between 2010 and 2018 were identified as described previously [4]. So-called ‘en plaque’ meningiomas, infratentorial meningiomas, and patients who underwent surgery for recurrence were excluded. In line with a previous study that included meningiomas located in various intracranial locations [6], we defined ‘giant meningiomas’ as those with a largest diameter—in any plane—of 5 cm or larger, whereas non-giant meningiomas were smaller than 5 cm. The dural tail was not included in any tumor measurements.

In our center, follow-up of meningioma patients includes outpatient visits within 3 months postoperatively. Thereafter, the frequency of outpatient visits are individualized, and often include telephonic contacts, as we aim to limit the strain put on these fragile patients—many of which live rather distant from the hospital. Radiological follow-up is standardized with a first magnetic resonance imaging (MRI) within a few days after surgery, followed by another MRI after 2, 5 and 10 years for WHO grade I meningiomas, and yearly MRI follow-up for atypical meningiomas.

### Clinical and surgical data

Clinical data were extracted from the electronic patient files as described previously [4]. In brief, we included the following information: patient characteristics, preoperative physical status (Helsinki version of the American Society of Anesthesiologist (ASA) scale [9]), surgical indication, functional performance (Karnofsky Performance Status (KPS) scale [10]), surgical time, length of hospital stay, discharge location, histopathological diagnosis [10], and surgery-associated morbidity and mortality. In addition, we assessed pre- and postoperative independence, i.e. living at home. Postoperative independence was evaluated at discharge and within 1 year after the surgery. With regard to the independence (the rate of very old patients living at home 1 year after surgery) and postoperative functional performance (the KPS score), we reviewed the electronic health record data of every patient. For the KPS score estimation, we used only medical notes recorded during the visits in the outpatient clinic of the Department of Neurosurgery or Neurology, as both the neurological and performance status were well documented during these postoperative visits. If a patient had multiple visits in the outpatient clinic within the first postoperative year, we used the data of the last visit to estimate the 1-year postoperative KPS score. Since the time and frequency of postoperative visits of these very old patients varied throughout the study period, the time of the KPS score estimation also varied. Electronic health record data were also used to find out if operated patients lived at home 1 year after surgery. This electronic health record data also included data about the visits in other healthcare facilities than in the Department of Neurosurgery or Neurology. Therefore, the 1-year independence data is based on visits in any healthcare facility 1 year after surgery, whereas the KPS score estimation reflects the functional performance at the last known clinical visit in the Department of Neurosurgery or Neurology within the first postoperative year. For this reason, the KPS score follow-up time for the whole cohort is presented as a median follow-up time.

Surgical reports were reviewed to collect data on the tumor consistency (soft/intermediate/hard) and the extent of resection (partial/total). Postoperative complications were classified as minor or major based on the criteria applicable for craniotomy patients, as reported previously [11]. For mortality, we assessed in-hospital, 1-month and 1-year mortality rates. The nationwide patient data repository (Population Register Center) provided the information on any deaths during 1-year the follow-up.

### Radiological data

We used MRI data for radiological assessment of tumor diameter, volume, location, and peritumoral edema, as described previously [4]. The tumor diameter was measured in the axial, sagittal and coronal planes, and the largest recorded diameter was used to classify meningiomas into giants and non-giants. Tumor volumes, not including the dural tail, were calculated using the SmartBrush^®^ function of the neuronavigation software (Brainlab Elements^®^, Brainlab AG, Germany) using the T1-weighted + contrast sequence (1 mm slice thickness) data. Four tumor locations were defined: (1) convexity, (2) falx/parasagittal, (3) skull-base, and (4) other. For peritumoral edema, we calculated the edema index as the ratio between tumor and edema volume, and stratified it into three groups: (1) no edema, (2) moderate edema (the edema ray is smaller than or equal to the tumor diameter), and (3) severe edema (the ray of edema is larger than the tumor diameter) [12].

### Statistical analyses

Variables were analyzed as continuous, ordinal, or categorical. Categorical variables are presented as numbers with percentages and continuous variables as median ± interquartile ranges (IQR). Wilcoxon rank-sum, chi-square and Fisher’s exact tests were performed as appropriate. All variables were considered as non-normally distributed. Using univariate and multivariate models based on logistic regression analysis, we assessed associations of meningioma size (diameter increase), meningioma volume (volume increase), and giant size with outcome measures. Patients’ age, sex and preoperative independency were included in the multivariate model. The results of these analyses are presented as odds ratios (ORs) with 95% confidence intervals (CIs). As post-hoc analysis, we compared preoperative anticoagulant and antithrombic medication usage to postoperative intracranial hemorrhages (ICH) and the extent of tumor resection, tumor consistency, surgical time, complications and postoperative ICH to atypical histopathology. We used Stata version 16 (StataCorp, College Station, TX) for all statistical analyses. A p value of <0.05 was used to indicate statistical significance.

## Results

### Patient characteristics

Between 2010 and 2018, 76 very old patients underwent resection of a supratentorial meningioma, including 28 giant and 48 non-giant meningiomas. In both groups, the median age was 83 years and the majority of patients were females (female:male ratio is 2:1) (Table 1). Cognitive impairment was the most common indication for surgery, particularly in patients with giant meningiomas (Table 1). Based on the Helsinki ASA score, nearly all patients in both groups suffered from severe or unbalanced systemic diseases or were clearly symptomatic due to the meningioma (Table 1). The median preoperative KPS scores (60) and the preoperative rate of patients living at home was comparable between non-giant and giant meningioma patients (Table 1).

**Table 1:** Patient, meningioma and surgical characteristics

	Non-giant (<5.0 cm)	Giant (≥5 cm)	p Value
*Preoperative characteristics*			
N patients	48	28	–
Age, median (range)	83 (80–93)	83 (80–96)	0.54
Sex, n (%)			0.67
Men	16 (33)	8 (29)	
Women	32 (67)	20 (71)	
Surgical indication, n (%)			0.14
Cognitive impairment	14 (29)	14 (50)	
Hemiparesis/motor deficit	10 (21)	7 (25)	
Visual loss	5 (10)	4 (14)	
Seizure	5 (10)	0 (0)	
Balance disturbance	4 (8)	0 (0)	
Asymptomatic tumor growth	4 (8)	0 (0)	
Gait impairment	2 (4)	2 (7)	
Aphasia	2 (4)	0 (0)	
Other (headache, dermal effusion, hydrocephalus)	2 (5)	0 (0)	
Missing	0 (0)	1 (3)	
Helsinki ASA scale, n (%)			0.66
I	0 (0)	0 (0)	
II	4 (8)	1 (4)	
III	25 (52)	14 (50)	
IV	19 (40)	13 (46)	
V	0 (0)	0 (0)	
*Preoperative functional performance*			
Preoperative independency, n (%)			0.55
Independent	34 (71)	18 (64)	
Dependent	14 (29)	10 (36)	
Preoperative KPS, median (IQR)	60 (50–70)	60 (40–70)	0.36
*Meningioma characteristics*			
Meningioma size, median (IQR)	3.9 (3.3–4.3)	5.8 (5.5–6.6)	**<0.001**
Tumor volume (mm^3^), median (IQR)	15.7 (10.7–26.3)	56.7 (43.6–82.2)	**<0.001**
Missing, n (%)	2 (4)	1 (4)	
Meningioma location, n (%)			0.62
Convexity	20 (42)	11 (39)	
Falx	6 (13)	3 (11)	
Skull-base	19 (40)	14 (50)	
Other	3 (6)	0 (0)	
Edema index, n (%)			**0.04**
No	19 (40)	6 (21)	
Moderate	18 (38)	19 (68)	
Severe	11 (23)	3 (11)	
WHO grade diagnosis, n (%)			**0.02**
I	34 (71)	14 (50)	
II	7 (15)	11 (39)	
III	0 (0)	0 (0)	
Missing	7 (15)	3 (11)	
*Surgical characteristics*			
Skin-to-skin surgery time (min), median (IQR)	144 (113–192)	186 (137–242)	**0.04**
Extent of resection, n (%)			0.19
Partial	2 (4)	4 (14)	
Total	46 (96)	24 (86)	
Tumor consistency, n (%)			0.83
Soft	16 (33)	11 (39)	
Intermediate or various	4 (8)	4 (14)	
Hard	7 (15)	4 (14)	
Missing	21 (44)	9 (32)	
Length of hospitalization (days), median (IQR)	6.5 (5–8)	7 (5.5–8)	0.33

Bold represents the significant values

*ASA* American Society of Anesthesiologist, *IQR* interquartile range, *KPS* Karnofsky Performance Status, *WHO* World Health Organization

### Meningioma characteristics

The median maximum tumor size in patients with giant meningiomas was 5.8 cm, and the volume was 56.7 mm^3^ (Table 1). Meningiomas were most frequently located at the skull-base and convexity, regardless of tumor size (Table 1). Patients with giant meningiomas presented more commonly with peritumoral edema than non-giant meningioma patients (79% vs. 60%, p = 0.04). Severe edema was noted in only three patients with a giant meningioma (Table 1). Atypical histopathological diagnosis (WHO grade II) was reported in 11 (44%) and 7 (15%) (p = 0.02) of giant and non-giant meningioma patients, respectively (Table 1).

### Surgical characteristics

The median skin-to-skin surgical time was prolonged for giant meningiomas (186 vs. 144 min, p = 0.04, Table 1). Complete resection was accomplished in most (92%) cases, and the extent of resection did not depend on the tumor size (Table 1). The tumor consistency did not differ between the meningioma groups, although tumor consistency was not reported in the majority of cases (Table 1). No differences were found between groups regarding the length of hospital stay (Table 1).

### Functional outcome

**Fig. 1:**
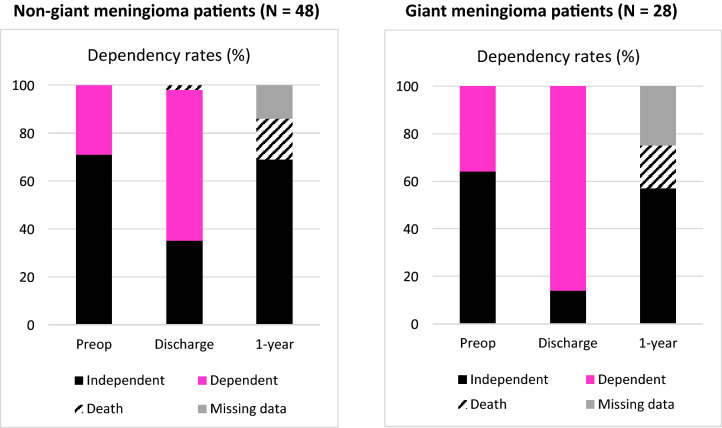
In this figure, the dependency rates are illustrated for the non-giant meningioma (left) and giant meningioma (right) patients. The preoperative dependency rates are compared to the corresponding rates at discharge and one year follow-up. The dependency rate at discharge clearly increased, in particular in the giant meningioma patients. At one year, the rate of patients living independent at home returned to the preoperative rate

Seventeen (36%) of the non-giant and 4 (14%) of the giant meningioma patients were discharged to their home (Fig. 1). One year after the surgery, the independency rates were 69% and 57% for non-giant and giant meningioma patients, respectively. After excluding the patients who died in the first year after surgery, 83% and 70% of the non-giant and giant meningioma patients, respectively, were living at home 1 year after the surgery (Fig. 1). There was a trend for patients with a giant size meningioma to have a reduced likelihood (OR = 0.30 (0.09–1.02)) to return home at discharge, but at 1 year after surgery. For the independence, the overall 1-year follow-up rate was 94% and 86% for the non-giant and giant meningioma patients (i.e. 6% and 14% of non-giant and giant meningioma patients, who were alive at 1 year, did not have any healthcare visits at 1 year).

**Fig. 2:**
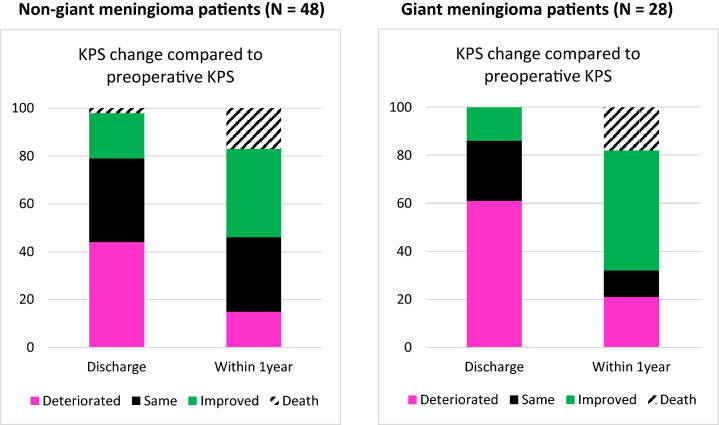
We present the change in functional performance, expressed as the Karnofsky Performance Scale (KPS), at discharge and within 1 year, while comparing to preoperative functioning. At discharge, a deterioration in KPS score was noted in 45% of non-giant meningioma patients (left figure), whereas a worsening in functional performance was noted in 61% of giant meningioma patients (right figure). With time, functional performance of both patient groups recovered as the majority of the patients returned to a similar or improved KPS score within one year

Compared to the preoperative KPS scores, a decrease in median postoperative KPS scores at discharge was noted in both patient groups, i.e. median KPS of 50 (IQR: 40–70) for non-giant meningioma and median KPS of 50 (IQR: 40–50) for giant meningioma patients. At discharge, a worsened KPS score was reported in 61% of the giant meningioma patients, compared to 45% in the non-giant group (Fig. 2). An increase in KPS at discharge was noted in a few cases of both patient groups (Fig. 2). When including the patients who died within the first year after surgery, the last median KPS scores reported within the first year was 65 (IQR: 0–80) for non-giant and 60 (IQR: 0–70) for giant meningioma patients. Among the surviving patients, the KPS score increased in 32 (51%) of all patients, and decreased in 13 (21%) patients. These KPS changes did not differ significantly between meningioma groups. The postoperative KPS score was estimated a median of 6 months after surgery.

### Postoperative mortality and morbidity

A total of five (7%) and 13 (18%) of all patients died during the first month and first year, respectively (Table 2). The mortality rates were similar between the non-giant and giant meningioma patients (Table 2) and not associated with meningioma diameter, volume or size (Supplementary Table 1). The follow-up data on 1-year mortality were complete.

**Table 2:** Postoperative complications for non-giant and giant meningiomas

	Non-giant (<5.0 cm)	Giant (≥5 cm)
*Postoperative complications*		
No complications, n (%)	23 (48)	10 (36)
One or more complications, n (%)	25 (52)	18 (64)
*Minor only, n (%)*	17 (35)	8 (29)
*Major only, n (%)*	8 (17)	10 (36)
Specification of complications, n (%)		
Major ICH (mass effect)	2 (4)	5 (18)
Minor ICH (no expansion)	8 (17)	6 (21)
Postoperative ischemic lesion	2 (4)	1 (4)
New hemiparesis	1 (2)	1 (4)
Other new neurological deficit	7 (15)	2 (7)
New epileptic seizure	4 (8)	4 (14)
Hydrocephalus	1 (2)	0 (0)
CSF leakage	0 (0)	2 (7)
Complication requiring secondary operation	3 (6)	5 (18)
Pulmonary embolism, DVT or sinus thrombosis	3 (6)	1 (4)
Pneumonia	1 (2)	3 (11)
UTI	7 (15)	6 (21)
Other infection	5 (10)	2 (7)
*Postoperative mortality*		
In-hospital mortality, n (%)	1 (2)	0 (0)
1-month mortality, n (%)	2 (4)	3 (11)
1-year mortality, n (%)	8 (17)	5 (18)

Overall complications were more commonly reported in patients with giant meningiomas (64%) compared to non-giant meningiomas (52%) (Table 2). Major complications were also more frequent in the giant meningioma patients (36% vs. 17%, p = 0.06) (Table 2). Postoperative ICH requiring a re-operation encompassed half (n = 5, 18%) of the major complications among the giant meningioma patients, but were noted in only two (4%) patients with a non-giant meningioma. In one patient with a non-giant meningioma, a shunt procedure was performed because of hydrocephalus. The overall complication rate was not associated with meningioma size or volume (Supplementary Table 1), whereas giant meningioma size showed a trend towards an increased likelihood to develop major complications (OR = 3.02 (0.96–9.50)).

## Discussion

This is the first study to report the functional outcome and complications of surgery of giant meningiomas in patients that are 80 years old and older. We found that patients with giant meningiomas were more likely to experience functional deterioration immediately following surgery. However, these differences attenuated with time, resulting in a comparable functional outcome 1 year after the surgery. Patients with giant meningiomas also suffered more often from major complications, particularly ICH. Interestingly, many giant meningiomas were classified as atypical.

### Functional outcome

We found that the discharge functional performance deteriorated in a large proportion (51%) of the very old patients, particularly in those with a giant meningioma (61%). These findings are in line with Dobran et al., who reported that a larger meningioma size (≥4 cm) is associated with 1-month KPS outcome in patients over 80 years of age [13]. At 1-year post surgery, median KPS score and independency rate were comparable to the preoperative performance status for giant and non-giant meningioma patients. This time-related postoperative recovery has been described in other studies as well [7, 12, 14]. In previous studies reporting postoperative KPS changes after 1 year, an improvement was reported in 41.2–86.5% of very old patients, and a worsening in 0–15.4% of patients [12, 13, 15, 16]. Our 1-year functional outcome results (overall KPS improvement: 51% and overall KPS deterioration: 21%) are in line with these results, although we found a somewhat higher rate in postoperative deterioration. This may be related to the relatively high complication rate in the giant meningioma patients.

### Postoperative morbidity and mortality

A larger tumor size has been associated with increased complication rates [12, 13, 15, 16]. In our series, overall complication rates were high (57%), and slightly higher (64%) in giant meningiomas. Previous studies on giant meningiomas have reported complication rates ranging from 46% to 59% [6, 7, 14, 17–19], which is in line with our results. However, the median age in our study was much higher than in the previous studies (Table 3).

**Table 3:** Characteristics of previous studies on surgery of giant meningiomas and rate of complications, postoperative ICH and atypical (WHO grade II) diagnosis

Study (author, year)	Location of meningioma	Average size (cm)	Population size	Mean age (years)	Complication rate	ICH rate (%)	Atypical diagnosis
Tomasello et al., 2003 [20]	Sphenocavernous	5.7	13	58	31%	0	N.S.
Behari et al., 2008 [21]	Sphenoid wing	6.1	20	47	75%	5	10%
Gazzeri et al., 2008 [22]	Olfactory groove	6.4	36	56	17%	0	0%
Romani et al., 2009 [7]	Olfactory groove	≥6	25	60	52%	4	N.S.
Tomasello et al., 2011 [17]	Olfactory groove	6.8	18	59	8%^a^	0	0%
Attia et al., 2012 [18]	Anterior clinoid process	5.9	22	54	59%	4.5	14%
Narayan et al., 2018 [6]	Supratentorial meningiomas	5.6	80	56	N.S.	2.5	20%
Champagne et al., 2018 [19]	Sphenoid wing	6.6	12	59	58%	0	17%
Li et al., 2020 [14]	Anterior skull base	6.1	70 (elderly)	72	46%	4.3^b^	24%

^a^Only visual deterioration reported

^b^Hemorrhagic and ischemic events combined

In our study, major complications were more frequent reporte in patients with giant meningiomas than in those with non-giant meningiomas (36% versus 17%, p = 0.06), predominantly due to postoperative ICHs (n = 5, 18%). Previous studies on meningiomas in very old patients reported major postoperative ICHs rates between 1.4% and 6.1% [3, 12, 23]; in patients with giant meningiomas, the rates were between 0% and 4.5% (Table 3) [6, 7, 14, 17–19]. The high postoperative ICH rate in our series (18%) is probably due to the combination of the very old age and giant meningioma size. In very old patients, brain atrophy and decreased brain compliance may contribute to the risk of postoperative ICHs. A post hoc evaluation of the preoperative use of antithrombotic and anticoagulant medication showed that 17% of patients using anticoagulation developed a postoperative ICH, compared to 7% without anticoagulation. Interestingly, none of the patients who did not use antithrombotic medication prior to surgery developed ICH. In Helsinki, preoperative timing of discontinuation of anticoagulative drugs is determined individually based on indication, risk factors, drug type, dosage, and renal function. Antithrombotic medication is ceased a minimum of 5 days before cranial surgery. Despite the relatively high complication rate, the median length of hospital stay was only 7 days and unrelated to the size of meningiomas. Given the low rate of discharges to home, the relatively short length of hospital stay appears to be due to early transfers to smaller hospitals and healthcare centers.

Regarding mortality, a total of five (7%) and 13 (18%) patients died at 1-month and 1-year follow-up, respectively. Both of these mortality rates are comparable to previous studies of very old meningioma patients, which ranged from 0% to 23.5% [3, 12, 13, 15, 16, 23, 24] and 9.4% to 27.3% [12, 16, 24, 25], respectively. Based on our findings, giant meningiomas do not pose an increased mortality risk in highly selected very old patients.

### Histopathology of giant meningiomas

The rate of atypical (WHO grade II) histopathological diagnosis was high (44%) in giant meningiomas. A review of intracranial meningiomas in very old patients found rates of atypical histopathology varying from 10% to 32% [2]. In our reviewed series of giant meningiomas (Table 3), an atypical diagnosis was found in 0–24% of the cases [6, 7, 14, 17, 18, 20–22]. Recently, a larger tumor size has been associated with atypical histopathology [26–28]. Interestingly, the rate of atypical meningiomas reported in the literature has increased from 5% to 20–35% since the revised 2007 WHO criteria for histopathological grading of meningiomas has been applied [29, 30].

Atypical meningiomas tend to invade the brain [6, 31] and therefore complicate the surgery. In our series, neither tumor consistency, peritumoral edema, extent of resection, nor surgical time were associated with atypical histopathology. Although, atypical meningiomas were not associated with overall functional outcome or postoperative complication rate, the occurrence of postoperative ICHs requiring reoperation was 17% in atypical meningiomas, compared to 6% in benign meningiomas. We believe that our small cohort size may at least partly explain these negative statistical results.

Previous studies described more frequent and severe peritumoral edema in giant meningiomas and in tumors with atypical histopathology [3, 12, 13]. In our study, the presence of edema was more common in giant meningiomas, whereas severe edema according to the edema index was more common in non-giant meningiomas. The latter is probably related to the fact that edema was calculated as a factor of tumor size.

### Cognitive impairment in giant meningiomas

We found that cognitive impairment was the surgical indication in half of the giant meningioma patients, compared to 29% of the non-giant meningioma patients. Previous studies including very old patients who underwent meningioma surgery show conflicting results with regards to cognitive impairment. Some did not report cognitive symptoms [15, 16, 24], whereas others reported cognitive impairment in 51–59% of patients [7, 18, 19]. Previous studies on patients with giant meningiomas reported cognitive impairment as the surgical indication in 50–84% [32–34]. Based on our results, cognitive impairment is more common in giant meningiomas. Increasing meningioma size has been related to preoperative cognitive functioning [35]. On the one hand, this might be the result of larger tumors putting more pressure on the brain, thereby affecting cognitive functions more frequently and severely [36, 37]. On the other hand, this might be related to a delay in diagnosis in cognitively impaired patients. Indeed, a subtle onset of mild cognitive deficits is often ignored by patients, their relatives and physicians, or wrongly attributed to other factors like ageing or psychiatric diagnoses [38, 39]. During this delay, a meningioma may grow further while cognitive functions slowly deteriorate.

## Implications for clinical practice

We believe that a giant tumor size alone should not unequivocally argue against surgery in very old patients. However, particular attention should be paid to meticulous hemostasis, given the high rate of postoperative ICHs in these fragile patients. Furthermore, the high number of atypical giant meningiomas is noteworthy. Given the common invasive nature of atypical meningiomas, one could argue that surgeons should avoid resection of the invasive components of giant meningiomas in very old patients. This would leave the invaded cortex undisturbed and permanent neurological deficits would be less likely, thereby reducing the impact on functional outcome. Lastly, comprehensive and timely patient counseling is critically important for preventing further deterioration due to tumor growth during a wait-and-scan policy. Similarly, surgeons should discuss and anticipate postoperative rehabilitation related to a transient decrease in functional performance.

## Strengths and limitations

Our study has several strengths. Firstly, this is the first study to evaluate the effects of tumor size, i.e. giant versus non-giant, on surgical outcome and surgery-related morbidity and mortality in very old patients. Furthermore, we assessed the patients’ ability to return home after surgery. This measure can be determined rather easily in Finland, and also when using retrospective medical data. Our study also has limitations. For example, this retrospective study has a selection bias. Moreover, patients included in this study were derived from a single, high-volume academic center, limiting the external validity. In addition, this is a focused study on very old patients with giant meningiomas, thereby narrowing the applicability of the results to a small proportion of meningioma patients. As well, we were not able to assess the possible effect of postoperative radiotherapy on the functional outcome, and in particular on the cognitive performance. Therefore, this possible confounder needs to be addressed in future studies. Finally, since our follow-up period is only 1 year, we did not report radiological recurrence rates.

## Conclusion

Giant meningioma surgery in very old patients comes with an increased complication rate. This considered, the prevention of postoperative ICHs needs specific attention. Moreover, as atypical histopathology was common among the giant meningiomas, surgical strategies for giant meningiomas in very old patients should be planned accordingly.

## Supplementary information

Below is the link to the electronic supplementary material.

Supplementary material 1 (DOCX 17 kb)

## Data Availability

On request data is available for review.
