# Supplementary material for: Surgery on giant meningiomas in very old patients entails frequent postoperative intracranial hemorrhages and atypical histopathology
Source: J Neurooncol. 2021 Jan 21;152(1):195–204. doi: 10.1007/s11060-020-03693-4 (PMC7910228; doi:10.1007/s11060-020-03693-4)
Supplement: Supplementary file 1 — Supplementary material 1 (DOCX 17 kb) [file 11060_2020_3693_MOESM1_ESM.docx]

**Supplementary table 1.** Univariate and multivariate models (logistic regression analysis) of the associations between tumor size and postoperative outcomes and complications.

|  | Univariate,  ORs (95% CIs) | Multivariate*,  ORs (95% CIs) |
| --- | --- | --- |
| Discharge to home   - Per SD (1.4cm) increase of diameter - Per SD (31mm^3^) increase of volume - Non-giant - Giant | 0.61 (0.35-1.04)  0.53 (0.27-1.06)  *(Reference)*  0.30 (0.09-1.02) | 0.65 (0.36-1.15)  0.62 (0.30-1.30)  *(Reference)*  0.31 (0.09-1.10) |
| 1-year independency   - Per SD (1.4cm) increase of diameter - Per SD (31mm^3^) increase of volume - Non-giant - Giant | 0.81 (0.49-1.33)  0.79 (0.48-1.31)  *(Reference)*  0.73 (0.25-2.13) | 0.89 (0.47-1.67)  0.89 (0.48-1.65)  *(Reference)*  0.80 (0.20-3.14) |
| 1-month mortality   - Per SD (1.4cm) increase of diameter - Per SD (31mm^3^) increase of volume - Non-giant - Giant | 1.32 (0.55-3.17)  1.08 (0.41-2.88)  *(Reference)*  2.76 (0.43-17.63) | 1.26 (0.40-3.95)  1.03 (0.28-3.76)  *(Reference)*  3.90 (0.40-37.63) |
| 1-year mortality   - Per SD (1.4cm) increase of diameter - Per SD (31mm^3^) increase of volume - Non-giant - Giant | 1.23 (0.69-2.22)  1.13 (0.62-2.06)  *(Reference)*  1.09 (0.32-3.72) | 1.31 (0.58-2.96)  1.10 (0.52-2.33)  *(Reference)*  1.14 (0.23-5.58) |
| Complications (any)   - Per SD (1.4cm) increase of diameter - Per SD (31mm^3^) increase of volume - Non-giant - Giant | 1.18 (0.75-1.85)  1.11 (0.69-1.79)  *(Reference)*  1.66 (0.64-4.32) | 1.31 (0.80-2.13)  1.31 (0.76-2.27)  *(Reference)*  1.98 (0.70-5.57) |
| Major complications   - Per SD increase of size - Per SD increase of volume - Non-giant - Giant | 1.34 (0.79-2.25)  1.17 (0.69-1.98)  *(Reference)*  2.78 (0.94-8.21) | 1.33 (0.76-2.34)  1.18 (0.66-2.12)  *(Reference)*  3.02 (0.96-9.50) |

*Adjusted model included IM patients’ age, sex and preoperative independency.

CI=confidence interval; OR=odds ratio
